# Driver’s Facial Expression Recognition in Real-Time for Safe Driving

**DOI:** 10.3390/s18124270

**Published:** 2018-12-04

**Authors:** Mira Jeong, Byoung Chul Ko

**Affiliations:** Department of Computer Engineering, Keimyung University, Daegu 42601, Korea; mystroll24@gmail.com

**Keywords:** facial expression recognition, deep neural networks, embedded application, ADAS, weighted random forest

## Abstract

In recent years, researchers of deep neural networks (DNNs)-based facial expression recognition (FER) have reported results showing that these approaches overcome the limitations of conventional machine learning-based FER approaches. However, as DNN-based FER approaches require an excessive amount of memory and incur high processing costs, their application in various fields is very limited and depends on the hardware specifications. In this paper, we propose a fast FER algorithm for monitoring a driver’s emotions that is capable of operating in low specification devices installed in vehicles. For this purpose, a hierarchical weighted random forest (WRF) classifier that is trained based on the similarity of sample data, in order to improve its accuracy, is employed. In the first step, facial landmarks are detected from input images and geometric features are extracted, considering the spatial position between landmarks. These feature vectors are then implemented in the proposed hierarchical WRF classifier to classify facial expressions. Our method was evaluated experimentally using three databases, extended Cohn-Kanade database (CK+), MMI and the Keimyung University Facial Expression of Drivers (KMU-FED) database, and its performance was compared with that of state-of-the-art methods. The results show that our proposed method yields a performance similar to that of deep learning FER methods as 92.6% for CK+ and 76.7% for MMI, with a significantly reduced processing cost approximately 3731 times less than that of the DNN method. These results confirm that the proposed method is optimized for real-time embedded applications having limited computing resources.

## 1. Introduction 

Recognition of human emotion from images is an interesting research topic, the results of which can be implemented in facial expression recognition (FER). Currently, the results of research on automatic FER have been used in many applications such as human-computer interaction [1,2]; virtual reality (VR)- [3] and augmented reality (AR)- [4] based games [5,6]; customer marketing and advertising; education [7]; and advanced driver assistant systems (ADASs) [8]. In particular, FER is one of the most important factors of ADASs, because it can be used to detect driver fatigue and, in conjunction with the rapidly developing intelligent vehicle technologies, assist safe driving. Therefore, this paper is focused on FER that can facilitate safe driving by determining the psychological state of the driver using his/her facial expression (FE).

Although FER has been studied for many years in the computer vision, it still presents many challenges related to the complexity of facial expression; changes in facial pose and illumination conditions; and occlusions and variations between individuals in terms of attributes such as age, gender, ethnic background and personality. To overcome these challenges the research on FER approaches has proceeded in the following three research directions. 

The first FER approach consists of action unit (AU)-based methods. AUs are defined as the movement of the facial muscles and an FE is represented by the movements of several AUs. In AU-based methods, a system that is pre-trained to recognize AUs detects them in an input image and then determines the FE by means of decoding the detected AUs. However, as these methods are based on invisible micro muscle movements, it is difficult to detect AUs accurately using only the appearance information of the face. To detect many AUs correctly, these methods must include classifiers for each AU or use multi-label classification, which requires an advanced computing system [9,10].

The second FER approach utilizes feature representation. The processing in this approach is composed of three steps: face and facial component detection, feature extraction and FE classification. Expression classification utilizes pre-trained FE classifiers, such as support vector machines (SVMs) [11,12,13], AdaBoost [14] and hidden Markov models (HMMs) [15], to achieve the recognition results using the extracted features. Feature representation can also be divided into two categories: appearance features and geometric features. Appearance features describe the texture of the face using various feature descriptors, including a histogram of oriented gradients (HoG) [16,17,18], local binary pattern (LBP) [11,13,19,20,21], scale invariant feature transform (SIFT) [13,20], and Gabor filter-based texture information [1,19]. Geometric features describe the shape of the face or the position of the facial components. Appearance features yield a better performance than geometric features, however, geometric features are more robust to changes in face position, scale, size, and head orientation. 

In recent years, the third approach, deep neural networks (DNNs), has emerged as a general approach to machine learning, yielding state-of-the-art results in many computer vision studies that utilized the availability of big data [7]. In addition, improved results have been reported for DNN-based FER methods as compared to conventional FER methods because of their ability to construct discriminative features from learning tasks. In DNN-based FER methods, a variety of versions of DNN have been applied, such as convolutional neural networks (CNNs), long-short term memory (LSTM), generative adversarial networks (GANs) [1,22] and inception and ResNet modules [23], according to the applications in which they are to be implemented. DNN-based methods recognize FEs by combining detected AUs, rather than using overall facial features for FER [9,10]. For example, if a DNN detects some AUs from an image such as AU-1, AU-22, AU-25, and AU-26, the system will classify this image as expressing an emotion of the ‘surprise’ category.

Although very deep or wide networks-based FER approaches usually perform reasonably well, they still have a few problems related to processing time and memory consumption, which are associated with the multitudinous parameters in the training and inference processes. However, in most embedded systems, including intelligent vehicle systems, real-time processing of DNNs is a heavy burden. Therefore, instead of a DNN, we propose an FER algorithm based on the proposed hierarchical weighted random forest (WRF) classifier that is capable of operating in low specification devices while achieving a comparable FER performance with a fast speed. 

The remainder of this paper is structured as follows. We present an overview of the related work on FER in Section 2. Section 3 provides the details of our proposed method in terms of feature extraction and the classifier. Section 4 provides a comprehensive evaluation of the proposed method through various experiments. Finally, the paper is concluded in Section 5.

## 2. Related Work

Automated FER methods have been widely studied for many years [7]. Because the most important factor that determines an FER method’s performance is the use of the most discriminative features, they can be divided into two categories, those using hand-crafted features and those using features generated by a deep learning network.

The first hand-crafted features include appearance and geometric features. As mentioned in the Introduction, HoG, LBP, SIFT and the Gabor filter are frequently employed as appearance feature descriptors. Chang and Chen [24] recognized FE by combining different AUs that were used for describing the basic muscle movement of a human face. This method used the input vector that is composed of facial characteristic points movements and two different neural network-based expression classifiers including a radial basis function network and a multilayer perceptron network.

Zavaschi et al. [19] proposed a novel FER scheme that employs a combination of the Gabor filter and LBP and SVM classifiers. Then a multi-objective genetic algorithm is used to search for the best ensemble using as objective functions the minimization of both the error rate and the size of the ensemble. Greche et al. [18] presented an FER based on three steps consisting of data preparation, features extraction using HoG and template matching for classification using normalized cross correlation. In this method, experimentation was carried out on CK+ datasets, and it gave 83.6% accuracy on five FEs. Carcagni et al. [13] reported a comprehensive study on the application of the HoG descriptor with an SVM classifier in FER. Luo et al. [21] used the LBP appearance features with principal component analysis and an SVM classifier for FER. 

In FER methods, the geometric features are defined using the locations and shapes of facial components extracted from an input image, and the relationship between related facial components is considered [25]. Therefore, most geometric feature-based methods include the major step of detecting facial components using the active appearance model (AMM) [26] or the active shape-based model (ASM) [27]. Choi et al. [28] proposed a technique for real-time recognition of FE which uses the AAM [26] with second order minimization and a neural network. The high dimensional feature vectors, which consist of a facial shape and texture, can be handled by a multi-layer perceptron model. Tanchotsrinon et al. [29] presented a graph-based feature extraction method that consists of three parts:(1) locating 14 points in the face region to extract graph-based features, (2) composing the graph-based features defined by the Euclidean distances for edges connecting the 14 points and (3) recognizing FE using neural networks with corresponding feature vectors. Suk et al. [12] presented real-time FER for use in a mobile application. This method first extracts the neutral features from a neutral face detected by an SVM and the mouth status. While it continues to update neutral features, this method generates new dynamic features using the displacement between the most recent neutral feature and the current feature if the face is recognized as having a non-neutral expression. Finally, it returns the recognized resultant expression by SVM classifiers and the dynamic features. This method showed experimental results with 86% of accuracy with 10-fold cross validation in 309 video samples of the extended Cohn-Kanade Dataset CK+ dataset [30]. Perikos et al. [31] recognized FE using adaptive neuro fuzzy inference systems. In this method, FEs detect facial deformations of specific regions such as eyes, eyebrows and mouth, and extract characteristics such as locations, length, width, and shape. Then, the feature vectors representing the deformations of the facial expressions are applied to adaptive neuro fuzzy inference systems to recognize FEs. This method showed approximately 90% average accuracy for Japanese female facial expressions (JAFFE) [32].

In summary, conventional feature extraction-based methods are suitable for real-time embedded systems because they can quickly learn and they operate effectively with a small amount of data; however, in terms of performance, they are inferior to DNN methods.

Recently, the use of features generated by deep learning networks has become the approach most widely used in studies on FER. Mollahosseini et al. [33] proposed a new DNN architecture for FER consisting of two convolutional layers, each followed by max pooling, and then four inception layers. The network of this method is a single component architecture. So, it takes registered facial images as the input and classifies them into either of the six basic or the neutral expressions. This method showed experimental results with 93.2% accuracy for CK+ and 77.6% accuracy for the MMI database [34,35]. In addition, Hasani et al. [23] presented 3D convolution networks (3D CNNs) that consist of 3D Inception-ResNet layers followed by an LSTM unit that together extract the spatial relations within facial images, as well as the temporal relations between different frames in the video. Facial landmark points are also used as inputs to the network which emphasizes the importance of facial components rather than facial regions that may not contribute significantly to generating FEs. Liu et al. [36] combined deep metric loss and softmax loss in a unified framework with two fully connected layer branches to alleviate the attribute variations introduced by different identities. A generalized adaptive (N+M)-tuple clusters loss function together with an identity-aware hard-negative mining and online positive mining scheme were proposed for identity-invariant FER. 

Recently, generative adversarial networks (GANs) have shown successful results achieved by means of a two-player game between a generator G and a discriminator D. Yang et al. [1] proposed the de-expression residue learning (DeRL) method which has two learning processes: (1) learning for the neutral face generation performed by conditional GANs, and (2) learning from the intermediate layer of the generator to classify FEs. This learning procedure can capture the expressive component of FEs that were recorded in the generative model. Zhang et al. [22] proposed a deep learning model in which different poses and expressions are utilized jointly for simultaneous facial image synthesis and pose-invariant FER based on GANs. The proposed GAN model automatically generates face images with different expressions under arbitrary poses to enlarge and enrich the training set for FER. Quantitative evaluations on Multi-PIE [37] and Static Facial Expressions in the wild (SFEW) [38] datasets had 91.8% accuracy for the Multi-PIE and 26.58% accuracy for the SFEW dataset. 

Unlike the above approaches that use overall face features, AU-based methods detect pre-defined AUs and then decode specific expressions from the Facial Action Coding System (FACS). Recently, AU-based methods have been applied to the deep learning approach. Zhao et al. [9] constructed deep region and multi-label learning to detect AUs and recognize FEs by dividing the aligned face images into 8 × 8 patches. This system showed a high AU detection performance which was achieved by considering the correlations between AUs; however, the results relied on the face alignment, and treating all blocks equally may degrade the importance of some regions. Liu et al. [10] proposed AU-inspired deep networks (AUDNs) to explore a psychological theory that expressions can be decomposed into multiple facial AUs. An AUDN consists of three processes: (1) a convolutional layer and a max-pooling layer to learn the micro-action-pattern (MAP) representation, (2) feature grouping to integrate correlated MAPs to produce mid-level semantics and (3) a multilayer learning process to construct sub-networks for higher-level representations. The performance evaluation was performed on seven expression categories including neutral using average accuracy, and it gave the 93.7% accuracy for CK+ and 75.85% for MMI database. 

Although DNN-based FER is one of the most recently developed methods and achieves outstanding results, this approach still requires an excessive amount of memory and incurs high processing costs as the network is deep and wide. Therefore, conventional classification algorithms are still being studied for implementation in real-time embedded systems because of their low computational complexity and high degree of accuracy [7].

**Figure 1:**
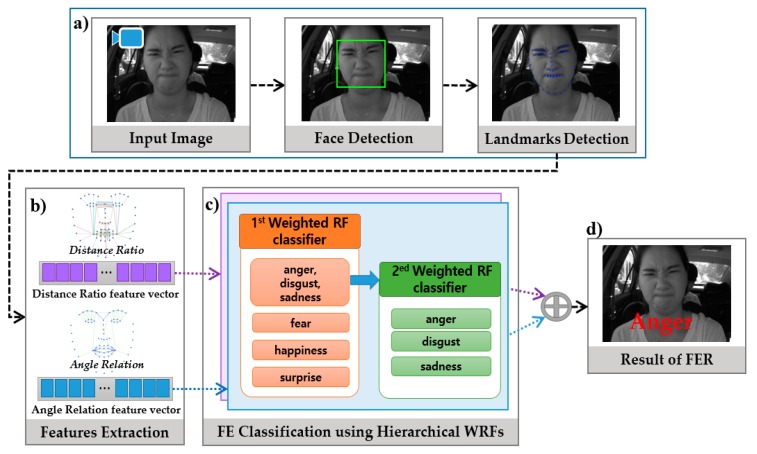
Overview of the proposed method for facial expression recognition. (**a**) the face region and facial landmarks are extracted from the image; (**b**) two geometric features are extracted based on the distance ratio and angle relations; (**c**) the hierarchical weighted random forest classifies the facial expression (**d**).

In the early versions of this paper [25], the feasibility of implementing FER in an embedded system using a simple hierarchical random forest (RF) was demonstrated. However, unlike in Reference [25], we introduce a new hierarchical structure that is constructed according to the similarity of facial expression and a new algorithm for constructing WRF, as shown in Figure 1. The major contributions of this paper are as follows:

- To generate the optimal split function of a tree, we propose using data similarity for information gain instead of entropy.
- We improve the classification performance by changing the hierarchical structure of the classifier and improve the WRF instead of using a general RF.
- In experiments in which the results of our study were compared with those of state-of-the-art studies using various benchmark databases, the proposed method shows good performance with a fast speed.
- The proposed method is successfully applied to the database consisting of images captured in an actual driving environment, and we confirm that its FE accuracy is high despite changes in various external environments.
- Through the proposed FER method, we show the possibility to apply the proposed method to the embedded systems such as intelligent vehicles entertainment, education, virtual reality, and games without sacrificing accuracy.

## 3. Facial Expression Recognition Approach

### 3.1 System Overview

To reduce the burden of feature extraction in a real-time system, in our method we use compact features reflecting the facial micro movement together with a fast and efficient classifier. For feature extraction, we propose a concise geometric feature descriptor based on the spatial relations between important face locations using the distance ratio and angle relations. For FE classification, we propose the new hierarchical WRF classifier that is composed of an ensemble of decision trees to learn the dynamic variation of FEs. 

The major steps of this paper, together with an overview of the procedures of the method, are shown in Figure 1. First, the facial region and landmarks are detected in an input image using the face and landmark detector of DLib [39] (Figure 1a). DLib is an open source machine library that provides a face detector and landmark detectors. After face detection, the trained facial landmark detector of DLib is used to predict the location of 68 (*x*, *y*) -coordinates that map to facial structures on the face. Second, the geometric features are constructed based on the spatial relations such as distance ratio and angle relation between some specified facial landmarks (Figure 1b). The FE is recognized using the hierarchical WRF which is hierarchically constructed according to the dissimilarity of FE groups as shown in Figure 1c. The first WRF classifies fear, happiness and another expression group and the second WRF classifies anger, disgust and sadness from the other group to achieve a more precise classification. The final probability of an FE class is estimated by combination of each WRF’s probability (Figure 1d). In this study, we evaluated the performance of our proposed algorithm using the well-known extended Cohn-Kanade (CK+) [30], MMI [34,35] and the Keimyung University Facial Expression of Drivers (KMU-FED) databases which include six basic expressions (anger, disgust, fear, happiness, sadness, and surprise).

### 3.2. Geometric Features 

To recognize the facial expression in real time with limited computing resources, we use geometric features, which require a lower processing cost than appearance features such as HoG or LBP features. General geometric features for FE describe the shape of the face or the spatial relations between its components. However, because they can change owing to the face rotation or scaling, to complement the spatial relations we use the distance ratio and the angle relations between relative positions of landmarks that are robust to face rotation and scaling. 

**Figure 2:**
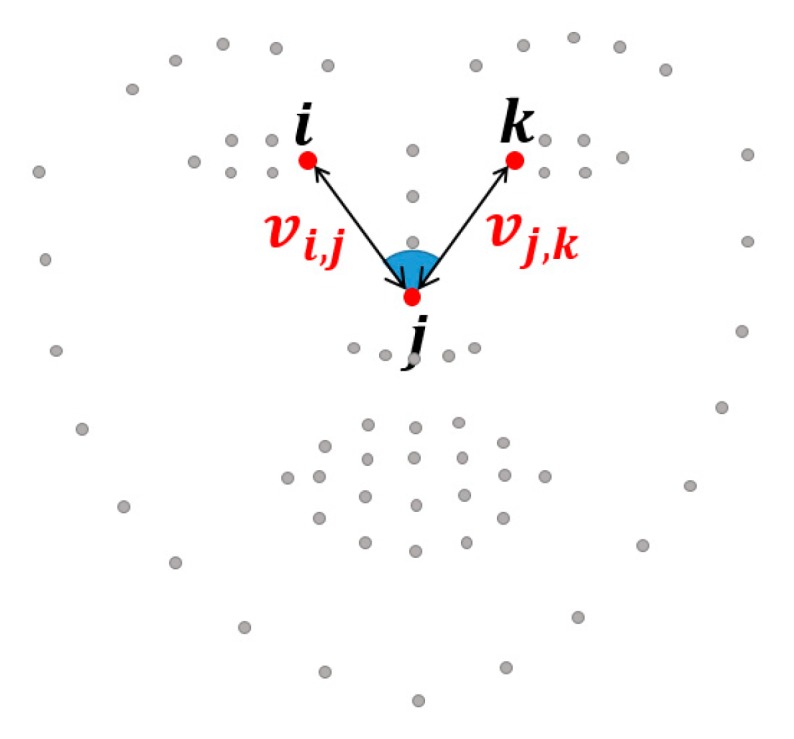
Geometric features using the spatial relations among three landmarks {*i*, *j*, *k*} such as the distance ratio and angles relations.

As the distance ratio feature, we define two individual vectors *vi, j* of the pairs of landmarks {*i, j*} and *vj,k* of the pairs of landmarks {*j, k*}, as shown in Figure 2. The spatial distance ratio is calculated using the two vectors to complement the spatial relations which can change as a result of face rotation or face scaling:

(1) Distratio=vi, jvj,k

The angle feature between three landmarks is extracted, as shown in Figure 2. The angle feature of the three landmarks {*i, j, k*} is modelled as the angle relations:

(2) Anglerelat=θ(vi,jvj,k)

where *v_a,b_* and *v_b,c_* are vectors that point from landmark *a* to landmark *b* and landmark *b* to landmark *c* respectively. The distance ratio and angle relations are sufficiently robust to changes due to face rotation or face scaling. 

**Figure 3:**
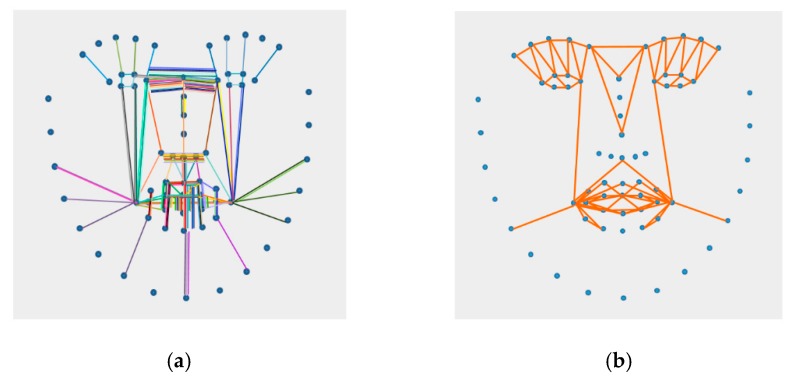
Landmark sets for defining the geometric feature descriptor. (**a**) Landmark set for the distance ratio and (**b**) landmark set for the angle relations.

An accurate feature descriptor should describe the features that discriminate various facial expressions using as many landmarks as possible. However, some landmarks may even degrade the FE classification performance. Therefore, we define influential landmarks that are located around the mouth, chin and eye region to compose discriminative feature vectors for FEs, as shown in Figure 3. By using a limited number of landmarks instead of all the landmarks, the proposed algorithm is able to achieve a reduced processing cost as well as improved accuracy. As shown in Figure 3, we extract 84 dimensional distance ratios and 88 dimensional angle relations. These features are inputs to the WRF classifier.

### 3.3. Facial Expression Classification 

#### 3.3.1. Random Forest Classifier

An RF classifier is an ensemble learning method consisting of a number of decision trees, where each tree is randomly grown with bootstrap aggregating or bagging in the training process [34]. Because an RF is based on randomizing techniques with regards to subset and feature selection while growing the trees, it is known as a classifier that is robust to overfitting, and it generates a better performance than SVM or AdaBoost-based methods [40,41]. 

In the training task, an RF decision tree extracts a subset S from the training sample data using bagging. A binary decision tree is grown in a top-down induction, beginning with the root node. At the *i*-th, node a subset $Q_{i}$ is split into subset $Q_{i}^{L}$ and $Q_{i}^{R}$ by the split function f(v) consisting of randomly chosen feature vectors v and a threshold τ. The feature vectors and threshold value are repeatedly created to determine an optimal split function. From among these, we choose an optimal pair composed of a split function and a threshold that maximizes the information gain about the corresponding node. This node split process is repeated until the maximum depth is reached or the information gain is zero. At the end of this iteration, a leaf node has posterior probability and class distribution p(c|l) for each class. 

In the test process, sample data are inputs to all the trees of the trained RF classifier and then they reach the leaf nodes of each tree. The final class distribution is generated by the ensemble of each distribution of all the leaf nodes $L=\left(l_{1},\;l_{2},\;\ldots ,\;l_{T}\right)$. $c_{m}$ is selected as the final class $\hat{y_{i}}$ of the input sample if the final class distribution $p(c_{m}|L)$ has the maximum value:

(3) $$\hat{y_{i}}=\arg\begin{array}{c} max\\ c \end{array}\left\{\frac{1}{T}\overset{T}{\underset{t=1}{\sum }}P(c_{i}|l_{t})\right\}$$

#### 3.3.2. Data Similarity for Information Gain

In a conventional RF classifier, one decision tree is generated in a top-down manner starting from the root node. The sample data of a parent node are separated into two subsets of child nodes based on the optimal split function among several candidate split functions. The process of selecting the optimal split function resembles searching an optimal information gain value that is calculated by the entropy of the subsets of two child nodes. In general, entropy is used to evaluate information gain, which is a method that uses the class distribution for sample data in each node. However, as the entropy-based method for searching the optimal split function does not take into account the characteristics of the values of the sample data but reflects only the class distribution, the classification accuracy can be degraded for data that have similar types of feature vectors for some classes. In our distance ratio and angle relations-based feature vectors, we can observe that the data distribution within each class is similar, but some classes even have a similar distribution for the feature vector included in different classes.

In this study, we improved the classification accuracy by means of using a node splitting process that considers the data similarity of the feature vectors. In other words, we can group similar sample data in the current node using the data similarity of feature vectors in the node splitting process. As a result of repeating this splitting process until it reaches the leaf nodes, the leaf nodes contain similar data; this approach is naturally a means of creating a tree that can classify several classes. Although there are two or more classes that have similar feature vectors in a leaf node, appropriate test results can be obtained by using the class distribution in the leaf node. Therefore, by splitting a node based on the data similarity, this decision tree can provide a very good performance for not only discriminative but also non-discriminative input data among classes.

To construct a decision tree based on data similarity, the data similarity is extracted from variances of values for each dimension of the feature vector, instead of using entropy as in the general RF classifier.

At an *i*-th node, a subset $Q_{i}=\{\left(x_{j},\;\;y_{j}\right)|j=1,2,\;\ldots .N\}\;$ is split into subsets $Q_{i}^{L}=\{\left(x_{l},\;\;y_{l}\right)|l=1,2,\;\ldots .N_{L}\}$ and $Q_{i}^{R}=\{\left(x_{r},\;\;y_{r}\right)|r=1,2,\;\ldots .N_{R}\}\;$ by split function f(v) consisting of randomly chosen feature vectors v and a threshold value τ. For *i*-th node splitting, we select a split function with the maximum information gain from among several candidate split functions. The information gain $\Delta G_{i}$ is easily calculated through the data similarity $S_{i}$, $S_{i_{l}}$, $S_{i_{r}}$ of each sample data item in the *i*-th node and the left (*l*) and right (*r*) child nodes:

(4) $$\Delta G_{i}=\overset{N}{\underset{j=1}{\sum }}S_{i}-\left(\frac{\left|N_{L}\right|}{\left|N\right|}\cdot \overset{N_{L}}{\underset{l=1}{\sum }}S_{i_{l}}+\frac{\left|N_{R}\right|}{\left|N\right|}\cdot \overset{N_{R}}{\underset{r=1}{\sum }}S_{i_{r}}\right)$$

where N indicates the number of the subsets $Q_{i}$ of the training data arriving at Node *i* and $N_{l}$ and $N_{r}$ are the number of data of the left and right split nodes, respectively. The data similarity $S_{l}$ is measured using the between-class variance of the subset belonging to an arbitrary node. To achieve this, we first estimate the between-class variance $B{\left(i\right)}_{var}^{f}$ for the *f*-th feature dimension in the *i*-th node using:

(5) $$B{\left(i\right)}_{var}^{f}=\overset{C}{\underset{c=1}{\sum }}\frac{\left|Q_{i}^{c}\right|}{\left|Q_{i}\right|}\cdot {\left(\mu^{f}-\mu_{c}^{f}\right)}^{2}$$

where c is the class index, $\left|Q_{i}\right|$ is the number of subsets $Q_{i}$ at the *i*-th node and $\left|Q_{i}^{c}\right|$ is the number of subsets $Q_{i}^{c}$ that belong to class *c*. $\mu^{f}$ and $\mu_{c}^{f}$ are the mean of all the values and mean of class *c* included in the *f*-th feature dimension, respectively. 

To evaluate the data similarity of all the feature vectors, we can use the sum of between-class variances of the respective dimensions:

(6) $$S_{i}=\overset{D}{\underset{f=0}{\sum }}B{\left(i\right)}_{var}^{f}$$

#### 3.3.3. Hierarchical Weighted Random Forest Classifier

As described in Section 3.3.1, the generalization performance of an RF classifier is good and its processing time is fast as it is based on a simple arithmetic operation in the test task. However, an RF depends on the number of decision trees and requires a certain amount of memory and CPU capacity. Therefore, boosted RF [42] and WRF [43] were introduced into the classification system to maintain the generality with a small number of decision trees when considering the fact that sequential training constructs complementary decision trees for the training sample [44].

**Figure 4:**
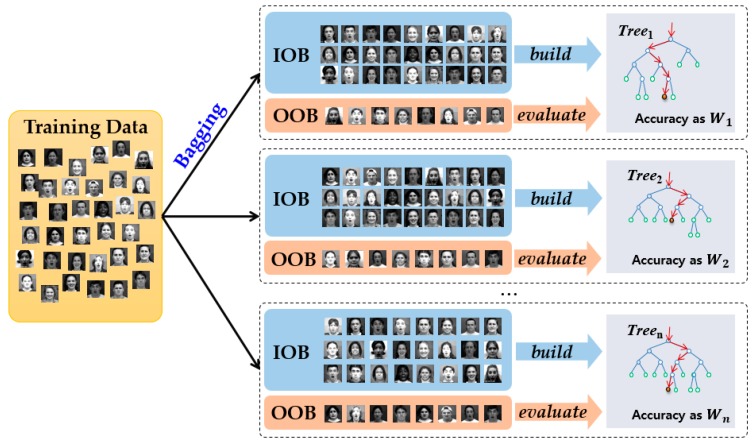
Training process of weight random forest using “in-of-bag” (IOB) and “out-of-bag” (OOB).

In the training task for the WRF classifier, the set of training sample data is divided into “in-of-bag” (IOB) and “out-of-bag” (OOB) through the bagging process, as shown in Figure 4. In the example of the first dotted box of Figure 4, a decision tree is built based on the sample data of the IOB, whereas an OOB subset is used to evaluate the classification ability of the tree learning from the IOB subset. If the accuracy of a decision tree is smaller than a permitted loss (threshold, 0.5), this tree is removed. However, if a decision tree is not removed, the weight of each tree is generated according to its accuracy, estimated based on OOB. Because OOB data are not involved in the building of the tree, the weight learning from this dataset can avoid over-fitting [45]. We can repeat the above procedure to generate the $T^{\prime }$ decision trees and the accuracy values of the remaining trees are utilized as the weights $w_{t}$. for each decision tree in the test task as:

(7) $$\hat{y_{i}}=\arg\begin{array}{c} max\\ c \end{array}\left\{\frac{1}{T^{\prime }}\overset{T^{\prime }}{\underset{t=1}{\sum }}w_{t}\cdot P(c_{i}|l_{t})\right\}$$

We employ the WRF classifier with data similarity to construct a feature that discriminates between several FEs. In this study, we learned of two types of WRF classifiers separately using two different feature vectors instead of aggregating them as one feature according to the experimental results presented in Reference [44]. We extract the feature vectors from a newly input image and input them into each corresponding classifier. Using the distance ratio and the angle relation vector, the probabilities of an FE class are computed by ensemble averaging of each probability distribution of all trees *L* = ($l_{1},\;l_{2},\;\ldots ,\;l_{T^{\prime }}$) using:

(8) $$P_{dist_{ratio}}\left(C_{FE}|L\right)=\frac{1}{T^{\prime }}\overset{T^{\prime }}{\underset{t=1}{\sum }}P(C_{FE}^{dist_{ratio}}|l_{t}).$$

(9) $$P_{angle_{relat}}\left(C_{FE}|L\right)=\frac{1}{T^{\prime }}\overset{T^{\prime }}{\underset{t=1}{\sum }}P(C_{FE}^{angle_{relat}}|l_{t})$$

Then, the final probability of an FE class is estimated by the weighted combination of each WRF’s probability:

(10) $$P\left(C_{FE}|L\right)=w_{1}\cdot P_{dist_{ratio}}\left(C_{FE}|L\right)+w_{2}\cdot P_{angle_{relat}}\left(C_{FE}|L\right)$$

The appropriate coefficient of weights $w_{1}$ and $w_{2}$ can be adjusted according to the characteristics of the FE data type. We set the weights $w_{1}$ and $w_{2}$ to 0.4 and 0.6, respectively, based on the experimental results. The probabilities of the two classifiers are combined by the linear weighted sum method to obtain the probabilities for each FE. After the overall processes of the two classifiers are complete, the class having the highest probability is determined as the final FE of the input image. The number of decision trees of each WRF is set to 200, which has been shown empirically to yield results and computation times that are comparable with those of related methods [41,44]. 

In this paper, we propose a WRF classifier with hierarchical structure to achieve more accurate classification. As shown in Figure 1, in the first level, the first WRF classifier is learned to distinguish between fear, happiness and another expression group. This is because the three emotions of anger, disgust and sadness have similar facial features and, therefore, they can be classified more precisely in the second level. In the second level, the second WRF classifies anger, disgust and sadness from the other group to achieve a more precise classification. The two types of WRF classifiers are learned separately using two feature vectors. The performance comparison used to prove the efficiency of hierarchical WRF is presented in experiment. 

## 4. Experimental Results

A number of databases for evaluating FER performance in image sequences have been used for comparative and extensive experiments. Among many FE-related databases, CK+, MMI, JAFFE, Facial Expression Recognition (FER)-2013 [46], and Karolinska Directed Emotional Face (KDEF) [47] composed of 2D images are the most frequently used databases in FER related studies. However, this paper aims at recognizing the FEs of the driver differently from other researches. Therefore, we conducted several comparative experiments on two well-known publicly-available FER databases, CK+ and MMI, to evaluate the effectiveness of the proposed method. Since there is no database for FER in the driving environment, we generated the KMU-FED database of images that captured the driver’s FE using a near-infrared (NIR) camera in a real driving environment. 

We first briefly describe the datasets used in the performance evaluation. Then we describe the results using these datasets in comparison with those of other state-of-the-art methods. As the evaluation measurement we used the accuracy, that is, the ratio of true outcomes (both true positive and true negative) to the total number of cases examined.

All the experiments were conducted using an Intel Core i7 processor with 8 GB of RAM running Microsoft Windows 10. In addition, all WRF approaches, including normal WRF and hierarchical WRF, were executed based on the CPU, and the DNN-based state-of-the-art approaches were executed based on a single Titan-X GPU.

### 4.1. Databases

(1) CK+ database 

The extended Cohn-Kanade database (CK+) [30] is the database most widely used in FER. This database contains 327 image sequences from 118 subjects and FE labels based on FACS. These image sequences start from the neutral state and end at the apex expression. All the sequence images include the facial landmarks, FACS code, and emotion labels. The emotion labels are categorized into seven emotions: anger, contempt, disgust, fear, happy, sadness, and surprise. In our experiments we used six emotions, omitting the contempt emotion, to allow a comparison of our method with other methods that are focused on six basic expression classes. We performed fivefold cross validation and measured the accuracy. The images have pixel resolutions of 640 × 480 and 640 × 490 with 8-bit precision for gray-scale values.

(2) MMI database

The MMI database [34,35] contains 213 image sequences, of which 205 sequences with frontal view faces of 31 subjects were used in our experiment. These image sequences start from the neutral state one of the six basic facial expressions, then go to the apex and end at the neutral state again. Since this database does not provide the location of the peak frame, we used three randomly collected peak frames with the provided six basic emotion labels. We also used the facial landmarks generated by Dlib, because this database does not provide actual positions of the facial landmarks. For the experiments, the database was divided into 10 groups for person-independent 10fold cross validation. We used the same evaluation method as for the CK+ database. The original size of each facial image is 720 pixels × 576 pixels.

(3) KMU-FED database

To verify the effectiveness of the proposed method in a real driving environment, we introduce a new benchmark dataset, called the KMU-FED database, for FER in an actual driving environment including problems that may occur on a real-life road. To construct the dataset, we captured benchmark dataset sequences in a real vehicle driving environment with an NIR camera. The KMU-FED database consists of drivers’ FEs captured using an NIR camera installed on the dashboard or steering wheel. It contains 55 image sequences from 12 subjects which include various changes in illumination (front, left, right and back light) and partial occlusions caused by hair or sunglasses. As when using the other databases, the cross validation method was used for algorithm evaluation when using KMU-FED. As no experimental results for the KMU-FED database from previous research studies exist, we measured and analysed only the accuracy of the proposed method. The images have pixel resolutions of 1600 pixels × 1200 pixels. The KMU-FD database of the full images is provided on our Webpage [48].

### 4.2. Facial Expression Recognition Performance Evaluation

To verify the effectiveness of the proposed FER method, we compared its performance with that of six state-of-the-art approaches that use either conventional algorithms or DNNs: (1) a real-time mobile FER [12] for a mobile application which uses dynamic features with an SVM classifier; (2) the AlexNets [49]-based FER approach which uses traditional CNN layered architecture; (3) a 3D CNN-based approach with deformable facial action part constraints (3DCNN-DAP) [36]; (4) a DNN that uses multiple inception layers [33]; (5) the Inception-ResNet (3DIR) network [23] which extends the well-known 2D Inception-ResNet module with LSTM; (6) an identity-aware FER that uses an adaptive deep metric learning as the (N+M)-tuple cluster loss [50]; (7) the proposed WRF which does not use a hierarchy structure (single-WRF); (8) the proposed hierarchical WRF with normal information gain (Proposed hierarchical WRF+Info.Gain), and the proposed hierarchical WRF with data similarity for information gain (Proposed hierarchical WRF+Data.Sim).

In Table 1, the two DNN-based methods, DNN [33] and Inception-ResNet and LSTM [23], produced a better FER performance than the other methods for the two datasets CK+ and MMI. However, as compared to the proposed algorithm, the performance difference is very low at 0.6% for CK + and 0.9–1.2% for MMI. The accuracy of AlexNets [49] and the 3DCNN-DAP [36] methods was lower than that of the proposed method by approximately 0.4% and 0.2% for CK+ and 20.7% and 13.3% for MMI, respectively. From the experimental results, we can see that the performance of our method (Proposed hierarchical WRF+Data.Sim) is similar to or better than that of a general shallow DNN, although it is slightly lower than that of a wide and deep DNN. Single-WRF’s performance is lower than that of hierarchy WRF, but its performance is better than that of the other shallow DNNs. Proposed hierarchical WRF+info.Gain has a 1.26% lower performance than Single-WRF (using Data.Sim), and 1.02% lower performance than Proposed hierarchical WRF+Data.Sim. From this result, we found that we can improve performance by using data similarity-based information gain rather than general entropy-based information gain to determine the split function of the tree node.

However, DNN-based methods are not suitable for low-specification systems such as intelligent vehicles because they require a lightweight algorithm that can run on CPUs instead of high-end GPUs to run in real time. Therefore, by means of additional experiments, we prove the efficiency of the proposed algorithm in terms of required memory (the number of parameters) and computational time (the number of operations).

### 4.3. Comparison of Parameter Numbers and Operations

In a real-time system, the number of parameters and the number of operations for a classifier are very important factors. Therefore, we compared the number of parameters and operations with the two DNN-based methods, two DNN model compression methods, and the proposed method (including feature extraction) using CK+ dataset. Among the DNN model compression techniques, MobileNet [51] was based on depthwise separable convolutions to reduce the number of parameters and operations. In this experiment, we set the width multiplier α as 0.5 and reduced image resolution ρ to 160 × 160 for MobileNet. The second method, SqueezeNet [52], employed three main strategies when designing CNN architectures such as replacing 3 × 3 filters with 1 × 1 filters, decreasing the number of input channels to 3 × 3 filters, and downsampling late in the network.

In this experiment, the proposed method was executed based on the CPU, and two CNN-based compression approaches were executed based on a single Titan-X GPU.

As shown in Table 2, it is confirmed that the proposed method requires a considerably smaller number of parameters and operations than general AlexNets [49] and a DNN-based method [33]. In particular, the number of parameters of the proposed method is approximately 244 times smaller than that of AlexNets [49] and the number of operations is 3731 times less than that of the DNN method [33]. The results show that although the accuracy of the proposed method is slightly degraded as compared to that of the deep and wide DNN-based method, the amount of computation and the amount of memory required are considerably smaller. 

In contrast, MobileNet [51] is 6.6% better than AlexNet [49] while being 45 times smaller and uses 9.4 times less computation than AlexNet. It is also 4% better than SqueezeNet [52] at about the same size and uses 22.3 times less computation. Compared with MobileNet [51] and SqueezeNet [52], the accuracy of the proposed method increased by 0.5–3.5%, but the number of parameters is about 5.3–5 times smaller and operations are 11,343–253,731 times reduced. Because the proposed method does not use all the parameters and it compares only a few specific nodes while growing the tree, the computation is very small.

From the results, we can confirm that the proposed method constitutes an optimized algorithm for recognizing the FE of a real-time embedded system such as an intelligent vehicle.

### 4.4. Expression Recognition Results

**Figure 5:**
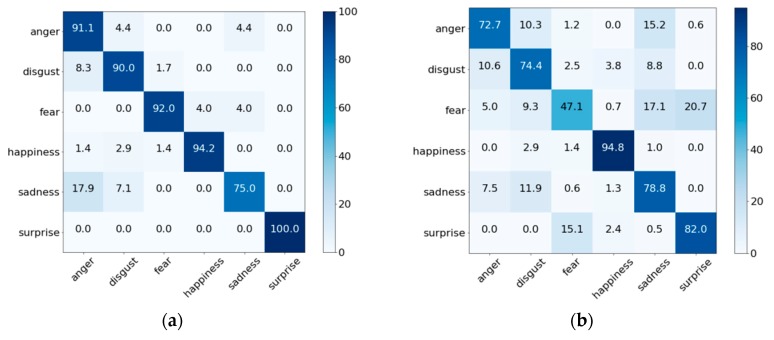
Confusion matrices of the proposed method using different databases (%). (**a**) CK+ database and (**b**) MMI database.

To determine whether the proposed method distinguishes each of the six FEs, we constructed confusion matrices for the CK+ and MMI databases, respectively, as shown in Figure 5. In Figure 5, (a) the highest performance was for surprise and the lowest performance was for sadness. The reason for this result is that the surprise FE change is relatively large, whereas the sadness has a similar FE with anger or disgust in the CK + database. In the case of the MMI database, the highest performance was for happiness and the lowest performance was for fear. The fear expression was frequently misjudged as the surprise or sadness expression, which is in contrast to the results in the case of the CK+ database. In summary, the classification performance for most of the FEs was similar, with the exception of the sadness expression in the case of CK+, and the fear expression in the case of MMI based on the confusion matrices. The main reason for the lower accuracy for these three FEs was that they involve similar movement of facial muscles or several important local features were lost because of faulty localization of landmarks.

**Figure 6:**
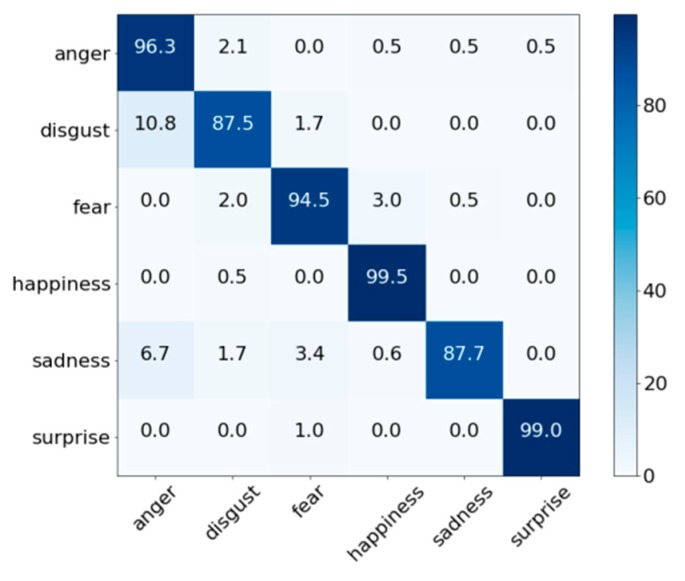
Confusion matrices of the proposed method using the KMU-FED database captured from a moving vehicle.

Figure 6 shows the confusion matrix of FE performance obtained using the proposed method and the KMU-FED database. Similar to the results in Figure 5, happiness has the highest performance at 99.5%. In contrast, the lowest FE performance was for disgust at 87.5%. Although KMU-FED was captured in an actual driving environment including problems that may occur in a real-life road, the overall accuracy yielded the best performance among the three databases at 94.7% versus 92.6% for CK+ and 76.7% for MMI. This is because each image was taken with an NIR camera and it has even brightness with a high resolution of 1600 pixels × 1200 pixels.

**Figure 7:**
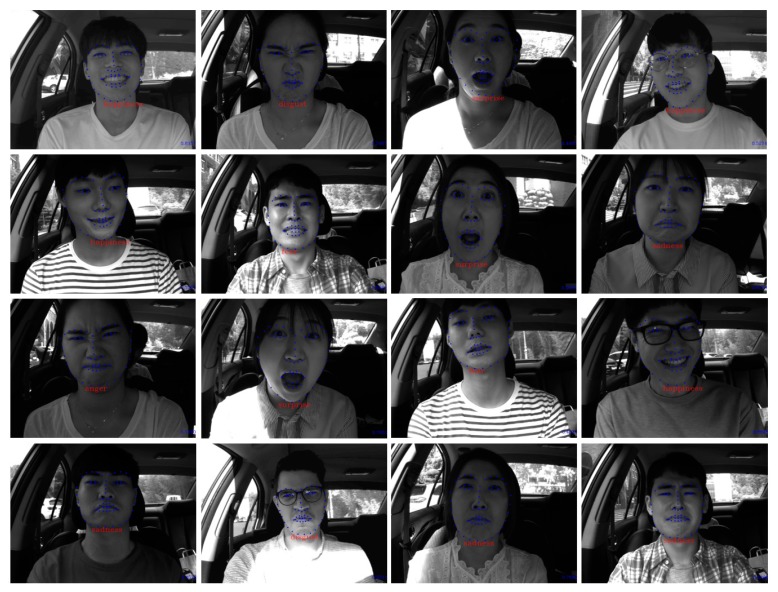
Facial expression recognition results in a moving vehicle using the KMU-FED database.

Figure 7 shows the FER results in a moving vehicle using the KMU-FED database. From the results, we know that the proposed algorithm recognizes FE correctly, although the intensity of the drivers’ image varies according to the degree of front, side and back sunlight, and in some images partial occlusions caused by hair or sunglasses are present. However, the proposed algorithm sometimes incorrectly recognized some FEs when landmark detectors lost the correct position because of fast face movements or abrupt changes of pose. Videos of the full results are provided on our Webpage [48].

## 5. Conclusions

In this paper, we presented a new FER method based on geometric features and the hierarchical WRF for real-time embedded systems, especially those of intelligent vehicles. As the initial step for building a real-time system, we first limited the number of landmarks used for generating geometric features instead of using all the landmarks. For the second step, we proposed a hierarchical WRF classifier to distinguish the FEs more precisely on two levels. In addition, because no appropriate dataset existed for FER that considers real outdoor driving situations, including the various illumination changes that occur, we generated a new benchmark dataset, KMU-FED, using an NIR camera to capture the images. A previous dataset was used as a reference. The experimental results show that the results of the proposed method without using a GPU are similar to those of the deep and wide DNN-based state-of-the-art FER approaches. Moreover, it was proved that the proposed method requires a low amount of memory and computing operations as compared to DNN-based approaches. Therefore, we confirm that the proposed FER method is applicable not only in the embedded systems of intelligent vehicles, but also in various other fields, such as entertainment, education, virtual reality, and games. 

In future work, we plan to improve our algorithm to reduce the false recognition rate when the face is rotated or partially occluded by objects. Moreover, a field test should be conducted with a programmed embedded board in a real driving environment. Finally, changes in facial expressions vary according to whether the subject is a child or an adult and according to the race of the subject, even for the same emotions; therefore, we will develop a new FER algorithm that can distinguish the FEs of varying subjects.

## Figures and Tables

**Table 1:** Comparison of the proposed method with state-of-the-art methods ^1^.

Comparison Methods	Accuracy (%)	
	CK+	MMI
Real-time mobile FER [12]	85.5	-
AlexNets [49]	92.2	56.0
3DCNN-DAP [36]	92.4	63.4
DNN [33]	93.2	77.9
Inception-ResNet and LSTM [23]	93.2	77.6
Adaptive Deep Metric Learning [50]	-	78.5
Single-WRF	92.2	70.9
Proposed hierarchical WRF+Info.Gain	90.9	69.7
Proposed hierarchical WRF+Data.Sim	92.6	76.7

^1^ Recognition performances of comparison methods are adapted from individual papers.

**Table 2:** Comparison of the number of parameters and operations for the proposed method and a deep neural network-based approach using the CK+ database.

Methods	Accuracy (%)	No. of Parameters (M)	No. of Operations (M)
AlexNets [49]	85.5	61	720
DNN [33]	93.2	9	25
MobileNet [51]	92.1	1.32	76
SqueezeNet [52]	89.1	1.25	1700
Proposed method	92.6	0.25	0.0067
